# Commercial feed containing porcine plasma spiked with African swine fever virus is not infective in pigs when administered for 14 consecutive days

**DOI:** 10.1371/journal.pone.0235895

**Published:** 2020-07-22

**Authors:** Elena Blázquez, Joan Pujols, Joaquim Segalés, Fernando Rodríguez, Joe Crenshaw, Carmen Rodríguez, Jesús Ródenas, Javier Polo

**Affiliations:** 1 IRTA, Centre de Recerca en Sanitat Animal (CReSA-IRTA), Barcelona, Spain; 2 APC EUROPE S.L.U., Granollers, Barcelona, Spain; 3 OIE Collaborating Centre for the Research and Control of Emerging and Re-emerging Swine Diseases in Europe (IRTA-CReSA), Bellaterra, Barcelona, Spain; 4 Departament de Sanitat i Anatomia Animals, Universitat Autònoma de Barcelona (UAB), Barcelona, Spain; 5 UAB, Centre de Recerca en Sanitat Animal (CReSA, IRTA-UAB), Campus de la Universitat Autònoma de Barcelona, Barcelona, Spain; 6 APC LLC, Ankeny, Iowa, United States of America; Plum Island Animal Disease Center, UNITED STATES

## Abstract

The objective of this study was to determine if commercially collected liquid porcine plasma contaminated with African swine fever virus (ASFV) and fed for 14 consecutive days would infect pigs. Commercially collected liquid porcine plasma was mixed with the serum from an ASFV experimentally infected pig. To simulate the potential of pigs slaughtered being ASFV viremic but asymptomatic and passing antemortem inspection, the ratio of liquid plasma from healthy animals to serum from an ASFV infected pig used in this study represented 0.4% or 2.0% of the pigs slaughtered being viremic (Studies 1 or 2, respectively). The contaminated liquid plasma was mixed on commercial feed and pigs were fed for 14 consecutive days providing to each pig 10^4.3^ or 10^5.0^ TCID_50_ ASFV daily (Studies 1 or 2, respectively). Pigs were observed for an additional 5 or 9 days (Studies 1 or 2, respectively). In both experiments, the pigs did not become infected with ASFV during the 14d feeding period or during the subsequent observation period. In these experiments, unprocessed liquid plasma contaminated with ASFV mixed on commercial feed and fed for 14 consecutive days did not infect pigs. From our results we can conclude that the infectious dose of ASFV on feed is much higher than that previously reported, at least with ASFV-spiked raw plasma.

## Introduction

African swine fever virus (ASFV) is an enveloped dsDNA virus of the *Asfarviridae* family [1] that can cause high mortality in pigs of all ages. Acute clinical forms of African swine fever (ASF) are characterized by high fever, loss of appetite, hemorrhages in the skin and internal organs, and death in 2–10 days. Mortality rates may be as high as 100%. The ASFV can be isolated from multiple organs, tissues and fluids from infected pigs and the disease must be reported to the World Organization of Animal Health (OIE).

Since the first ASF outbreak declared in Georgia in 2007, ASFV spread through the Caucasus. In Europe, the first notification of ASFV case in wild boar was reported by Lithuania in January 2014 and soon thereafter in Poland, Latvia and Estonia. From 2014 to present time, Ukraine, Romania, Hungary, Bulgaria, Slovakia, Czech Republic and Belgium have reported cases of ASFV in wild boar and some of these and other countries (like Greece) also reported cases in domestic pigs. All epidemiological data indicate that the EU has undergone repeated introductions of ASFV from the Eastern neighboring countries. The EU Reference Laboratory confirmed, through genetic sequencing of specific DNA fragments from virus isolates from Estonia, Latvia, Lithuania and Poland, that they all have the same origin as the strains that circulated in Belarus and Russia. Since August 2018, ASF cases have been reported in Asian countries including China, Vietnam, South Korea, North Korea, Philippines, Laos, Myanmar, Mongolia, Cambodia, and most recently in Timor-Leste, Papua New Guinea, Bali and Indonesia. ASFV is today a significant threat and trade restriction to the global swine industry.

Spray-dried plasma (SDP) is a dry protein ingredient containing a diverse mixture of many functional components such as immunoglobulins, albumin, fibrinogen, lipids, growth factors, biologically active peptides, transferrin, enzymes, hormones and other factors that have biological activity independent of their nutritional value [2]. SDP is extensively used in pig starter diets and consistently improves growth performance, and survival, especially under stressful conditions like pathogen challenge [3]. The beneficial effects of SDP are related to its modes of action that collectively support an efficient immune system [4]). Pigs and other animal models have been used to demonstrate that SDP improves intestinal barrier function, reduces intestinal inflammation and reduces the extent and severity of diarrhea [5–8]. Dietary SDP in pigs reduced the percentages of blood monocytes, macrophages in ileal Peyer’s patches and lymphoid nodes (LN), B lymphocytes and γδ+ T cells in LN, and intraepithelial lymphocytes as well as the density of lamina propria cells in the colon [9]. These data indicate that dietary SDP modulate functional and structural properties of the intestine. We speculated that maintaining intestinal barrier function, reducing inflammation and the number of monocytes and macrophages (target cells for ASFV) in Peyer’s patches and LN may reduce the infectivity of ASFV.

Contaminated feed and porcine origin feed ingredients have been considered risk factors for the spreading ASF. Dee et al. [10] demonstrated that ASFV can survive for extended time periods (30 days) under conditions of a transboundary simulation model. Niederwerder et al. [11] reported that a minimum infectious dose (MID) of 10^4^ 50% tissue culture infectious dose (TCID_50_) mixed into feed and administered as a single feeding was enough to infect 40% of exposed pigs. Extrapolating from this data the authors predicted that 10 exposures by feeding would result in 100% of the pigs becoming infected.

The prevalence of ASF globally has increased the fear that infected asymptomatic pigs may not be detected during antemortem inspection and could be slaughtered. Porcine origin feed ingredients could then be unknowingly contaminated with tissues from these pigs. Based on this premise and its potential impact on viral transmission, two studies were designed to determine if commercially collected unprocessed liquid porcine plasma (LPP) contaminated with blood from ASFV infected pigs then blended with feed (at infectious doses of 10^4^ or 10^5^ TCID_50_) daily for 14 consecutive days would be able to cause ASFV infection in naïve pigs.

## Materials and methods

### Ethical statement

For these studies, 30 Landrace x Lager White pigs (15 female and 15 male) were obtained from a commercial PRRSV-free farm with high sanitary status.

Animal procedures were approved by the committee of ethics and welfare “Comitè d’experimentació animal de la Generalitat de Catalunya” with the protocol approval number CEA 10604. The clinical state of the animals was evaluated by scoring the signs as a whole and according to severity, applying a score from 0 to 5: 0: no clinical signs, 1: mild pyrexia (39.6–40.0), 2: pyrexia (39.6–40.0) and mild clinical signs (skin, digestive), 3: pyrexia (40.0–40.5) and mild-moderate clinical signs (distal ear spots, mild limp, lying down, but remain alert), 4: pyrexia (40.5°C-41) and moderate clinical signs (remains stretched, only stands up when touched, hesitant step, subcutaneous bleeding <10%, diarrhea, mild tremors), 5: Pyrexia greater than 41 and other moderate-severe clinical signs (generalized subcutaneous bleeding, ataxia, spasticity, clouding, prostration, bloody diarrhea).

#### Ending points criteria

According to the results of the monitoring of clinical signs, the quantitative ending point criteria was applied: Score 0: Normality. No acting; Score from 1 to 3: Jointly propose investigating personnel of the procedure and veterinarian responsible for the application of a possible treatment. If a score of 4 is detected, the animal should be assessed for euthanasia. Score 5: Euthanasia.

#### Qualitative criteria

Euthanasia was applied if the animal had a temperature higher than 41.5°C without other signs, it was considered a qualitative sign of final point or serious signs such as if the pig is prostrated, unable to get up, or present lack of coordination of total movements or severe respiratory failure or coma.

### Inoculum preparation

Instead of SDPP, normally used in feed diets, we decided to use LPP to avoid the inactivation effect of the spray-drying process on ASFV [12]. Therefore, the use of non-treated LPP guaranteed high titer of live ASFV in the feed.

A 3 L batch of LPP was collected commercially from abattoirs under inspection of competent authorities and stored at 4°C before use. Three 10 mL sub-samples of LPP were stored at –80° C and later analyzed for absence of ASFV antibodies to exclude any potential cross-reaction with the inoculated ASFV and for the absence of ASFV Georgia 2007 genome by qRT-PCR.

Blood from an ASFV infected pig (Georgia 2007/1; [13]) was obtained after end-point euthanasia [14]. The blood was allowed to clot at room temperature for 2 h and stored at 4°C for 18 h. Serum was separated from the red blood cells by centrifugation for 15 min at 600 g; the supernatant was stored at -70° C until use. Serum aliquots (5 mL) were separated to measure the quantity of virus present in the serum. Titration was done on alveolar macrophages by a Immunoperoxidase monolayer assay (IPMA) method [15]. The serum contained ASFV 10^5.7^ TCID_50_/mL.

In Study I, 1,394.4 mL of LPP mixed with 5.6 mL of serum from the ASFV infected pig was the positive control achieving a final titer of 3.3 log TCID_50_/mL. This simulated the potential of 0.4% of the pigs slaughtered being ASFV viremic but asymptomatic and passing antemortem inspection. The negative control was 1.4 L LPP that did not contain serum from the ASFV infected pig. In Study II, 4,900.5 mL of LPP was mixed with 99.5 mL of serum from the ASFV infected pig achieving a final titer of 4.0 log TCID_50_/ml. This simulated the potential of 2.0% of the pigs slaughtered being ASFV viremic but asymptomatic and passing antemortem inspection.

### Inoculum in feed

A commercial mash nursery feed (Corp. Alim. Guissona, S.A., Lleida, Spain) was used. The feed contained animal fat and dicalcium phosphate derived from animal bone with no other animal source ingredients or feed (viral) mitigants declared on the label (S1 Table). A total of 100 mL of plasma (positive or negative control, respectively) was manually mixed with 1,000 g of feed. The mixture was prepared daily and stored at 4°C for 24 h to allow complete absorption of liquid into the feed.

### Study I

**Fig 1:**
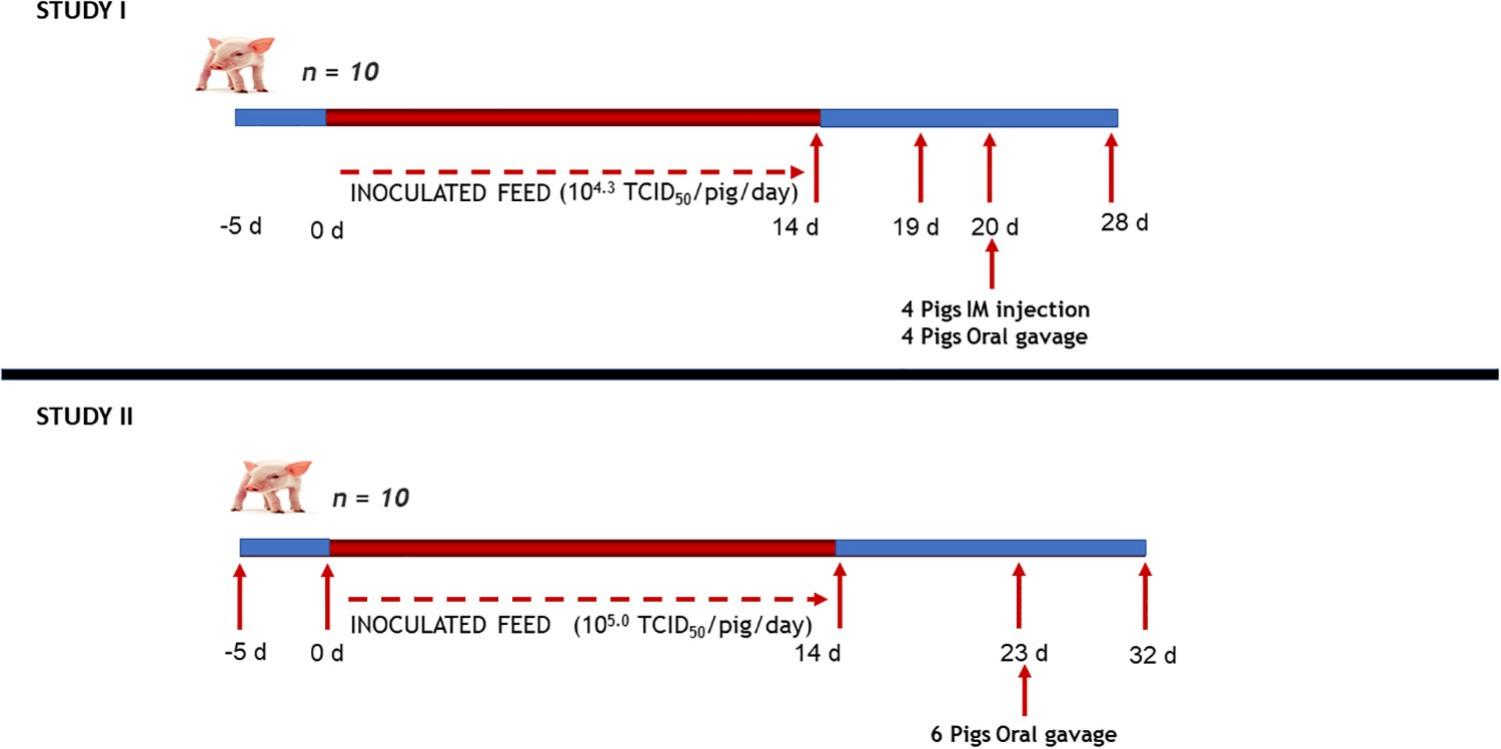
Chronological scheme of studies I and II. In the study I, at day 20, 4 pigs received IM injection of 10 mL Liquid ASFV inoculated plasma. Dose = 10^4.3^ TCID_50_/pig. Other 4 pigs received an oral gavage of 50 mL Liquid ASFV-inoculated plasma. Dose = 10^5.1^ TCID_50_/pig. In study II, at day 23, 6 pigs were oral gavaged with 50 mL Liquid ASFV-inoculated plasma. Dose = 10^5.7^ TCID50/pig.

Twenty naïve pigs weaned at 4 to 5 weeks of age divided into 2 groups of 10 pigs each were allocated in 2 separate rooms at IRTA-CReSA biosecurity level 3 animal facility and allowed a 5d adaption period. After an overnight fast, pigs were fed daily with 110 g of their respective treatment (either the positive or negative control feed) daily for 14 consecutive days (Fig 1). After the pigs consumed their treated feed, additional feed was provided ad libitum during the day and removed in the evening. After the 14-d feeding period, both groups of animals were provided non-inoculated feed and observed for signs of infection for 5 more days. On day 19, 2 pigs from each group were euthanized and necropsied. Because no fever or clinical signs were observed, Study I was extended to determine if the inoculum could be infectious by other routes of administration. On day 20, 4 of the positive control pigs were inoculated by intragastric tube (IG) with 50 mL of the LPP spiked with ASFV (single dose providing 10^5.1^ TCID_50_/pig), and the other 4 pigs from this group were injected intramuscularly (IM) with 10 mL of the ASFV inoculated LPP (single injection providing 10^4.3^ TCID_50_/pig). The same procedures were done for the negative control group, using non-inoculated LPP. Pigs were monitored for clinical signs daily for 8 days after IM or IG administration and all surviving pigs were euthanized on day 28 of the study.

### Study II

In Study II, only the positive control treatment was tested again with another group of 10 naïve pigs weaned at 4 to 5 weeks of age. The pigs were provided feed mixed with ASFV inoculated LPP as described above for 14 consecutive days, except that the concentration of ASFV administered daily on feed was 10^5.0^ TCID_50_/pig/day (Fig 1). There was a 9-d observation following the treatment period. On day 23, 6 pigs received 50 mL of ASFV inoculated LPP by IG (single dose providing 10^5.7^ TCID_50_/pig) to determine if the ASFV inoculated LPP contained infective virus. The remaining pigs were not administered IG gavage of the positive control LPP but remained the same pen as the IG pigs. All animals were euthanized on day 32.

#### Laboratory analysis

For Studies 1 and 2, rectal body temperatures and blood samples were taken on days 0, 2, 4, 7, 9, 11, 14, 19, 22 and 28. Viremia was determined by for real time PCR (qRT-PCR) analysis [16] and seroconversion was determined by ELISA (INgezim PPA COMPAC, INGENASA; Madrid, Spain). At necropsy, samples of tonsil, spleen, and retropharyngeal, submaxillary and gastro-hepatic lymph nodes were taken for subsequent qRT-PCR analysis. For each tissue, 0.1 g of tissue was homogenated using sterile PBS and TyssueLyser II (Qiagen, Hilden, Germany).

#### Confirmation of ASFV dose in the positive control feed

A retained frozen sample of 100 mL of the ASFV-spiked plasma from Study I was blended with 1000 g of feed, shaken and maintained at 4°C for 24 hours. A total of 20 g of inoculated feed were resuspended in 40 mL of PBS to evaluate infectivity potential of the inoculated feed. This solution was centrifuged twice at 3000 rpm for 15 minutes to clarify the supernatant. A titration of the supernatant was done in triplicate on porcine alveolar macrophages by a modified IPMA method [15]. Resulting in a titer of 3.96 ± 0.07 Log TCID_50_ ASFV in 100 g of feed.

## Results

Commercially collected LPP was tested and found to be free of ASFV genetic material and free from antibodies to ASFV.

All pigs administered feed mixed with LPP, both the positive and negative controls remained healthy for the 14-d treatment period and during the subsequent 5 or 9 d observation period (Studies I and II, respectively). No clinical signs of disease, fever, viremia or seroconversion was observed in any of these pigs. Tissue samples collected from the four pigs necropsied on day 19 of Study I (2 pigs from the positive and negative control) did not contain ASFV virus (negative qRT-PCR).

In Study I the 4 pigs injected IM with 10 mL of LPP inoculated with ASFV experienced 3 days post-inoculation high temperature (>41.5°C) and exhibiting clinical signs and were euthanized on day 23. One pig challenged with 50 mL IG of positive control LPP was experiencing high temperature and exhibiting clinical signs of infection and was euthanized 7 days later (day 27). The remaining three pigs were euthanized on day 28. All tissue samples of pigs receiving IM or IG with ASFV-spiked LPP became ASFV qRT-PCR positive (Table 1). These data confirm that the LPP containing serum from the ASFV infected pig contained infective virus. All pigs in the negative group of study I that received non-ASFV inoculated LPP by IM or IG were qRT-PCR negative in tissues and no seroconversion was observed in sera samples.

**Table 1:** qRT-PCR results (Ct value) of the different pigs necropsied during the study 1 in the positive control group.

Pig Id	Route	Day of necropsy	Temperature (C°)	Blood	Gastrohepatic node	Submaxillar node	Tonsil	Spleen
144	Feed	19	40.46	Neg.	Neg.	Neg.	Neg.	Neg.
158	Feed	19	40.38	Neg.	Neg.	Neg.	Neg.	Neg.
170	IM	23*	41.54	13.56	15.57	14.62	19.11	9.42
173	IM	23*	41.81	16.12	17.13	19.09	19.73	9.85
174	IM	23*	> 42	13.23	16.17	19.07	20.59	9.33
175	IM	23*	> 42	11.86	15.37	NA	17.30	9.43
141	IG	27*	41.89	8.75	10.44	11.42	11.00	9.71
147	IG	28	39.79	17.36	17.84	18.77	18.35	6.30
150	IG	28	41.19	19.81	13.15	15.16	14.63	7.66
160	IG	28	39.62	27.56	28.01	26.23	28.18	28.78

Feed: Administration of ASFV inoculated liquid porcine plasma mixed with the feed from day 0 to 14 of the study. IM: intramuscular injection of 10 mL of ASFV inoculated liquid porcine plasma on day 20. IG: intragastric tube administration of 50 mL of ASFV inoculated liquid porcine plasma on day 20. Neg.: Negative for ASFV genome by qRT-PCR. NA: not analysed.

*Pigs euthanized before the end of the experiment due to end-point criteria.

Study II, one pig died on day 22 with pericarditis, but without ASF clinical signs (Table 2) and blood, spleen, tonsils and lymph nodes were qRT-PCR negative for ASFV. For pigs given ASFV inoculated LPP by IG, one pig had qRT-PCR positive results in blood and tissues and 2 pigs had viral genome in blood or tonsils. This confirms that the LPP containing serum from the ASFV infected pig contained infective virus. For the 3 pigs not receiving IG, tissues from one pig was aRT-PCR positive for ASFV while two of the pigs did not become infected.

**Table 2:** qRT-PCR results (Ct value) of the different pigs necropsied during the study 2 in the positive control group.

Pig Id	Route	Day of necropsy	Temperature (C°)	Blood	Gastrohepatic node	Submaxillar node	Tonsil	Spleen
2	IG	32	39.5	Neg.	Neg.	Neg.	Neg.	Neg.
3	IG	32	40.12	Neg.	Neg.	Neg.	Neg.	Neg.
7	IG	32	39.92	Neg.	Neg.	Neg.	Neg.	Neg.
4	IG	32	39.89	Neg.	Neg.	Neg.	32.18	Neg.
1	IG	32	39.97	28.35	Neg.	Neg.	Ind	Neg.
10	IG	32	40.8	10.94	11.73	10.16	10.7	11.01
9	Feed	32	40.35	33.25	25.62	16.94	23.34	22.63
5	Feed	32	39.8	Neg.	Neg.	Neg.	Neg.	Neg.
6	Feed	32	39.56	Neg.	Neg.	Neg.	Neg.	Neg.
8*	Feed	22	NA	Neg.	Neg.	Neg.	Neg.	Neg.

*Pig #8 died on day 22 of study due to pericarditis. Blood and tissues were negative for ASFV at necropsy.

IG: intragastric tube administration of 50 mL of ASFV inoculated liquid porcine plasma on day 23.

Ind: indeterminate results. NA: not analysed. Neg.: Negative for ASFV genome by qRT-PCR.

## Discussion

The results of both studies during the feeding period demonstrated that LPP containing serum of an ASFV infected pig mixed on feed and fed daily to naïve pigs for 14 consecutive days at doses of 10^4.3^ or 10^5.0^ TCID/pig/day (Studies I and II, respectively) did not infect the pigs. In Study I, the ASFV inoculum used on the feed could be titrated close to the expected dose, confirming that the commercial feed used in both studies did not contain any ingredients or mitigants that could significantly impact recoverable viable virus in the feed. Our feeding study results contrasted with the 10^4^ TCID_50_ MID of ASFV reported for feed by Niederwerder et al. [11] and disagrees with the authors statistical model prediction that 10 consecutive exposures of this MID on feed would infect 100% of the pigs. Results of the present study suggest that the MID of ASFV on feed is higher than previously reported, at least when the feed is mixed with LPP contaminated with serum from an ASFV infected pig.

There were some experimental procedure differences between our feeding study and those reported by Niederwerder et al [11] that may account for the different results obtained. In the study conducted by Niederwerder et al [11] they added 10 mL of virus inoculum at a specified dose (ranging from 10^3^ to 10^8^ TCID_50_) to 100 g of feed in a bottle and allowed it to absorb for 30 s, then homogenized it by rolling and gently mixing the bottle by hand. Designated specific doses provided in only one feed exposure were used in 7 replications using 5 different pigs per replication. They used a plant-based feed of known formulation. Unfortunately, no data were reported about the virus concentration in the infected feed to confirm the dose administered for each replica. In the current studies, 100 mL of the inoculum was added to 1000 g of a commercial feed in a larger bottle, gently shaken and kept overnight at 4°C to allow a longer time for more complete absorption into the feed before feeding it. After an overnight fasting period, the group of 10 pigs were provided the feed at the same dose for 14 consecutive days. Also, in the current studies, ASFV was recovered from the feed after the overnight holding time confirming that the virus was infective at the time of feeding. Further the titration of virus on the feed confirmed the daily viral dose and confirmed that the feed did not contain a viral mitigant. However, our results showed the doses administered on the feed daily for 14 consecutive days were not infective, even though previous reports describe opposite results when fed at a similar dose [11]. Thus, it is possible that the difference between Niederwerder et al. [11] and the present studies resides in the 24-hour holding period affecting the ability of the virus to infect target sites in the pharynx during feeding.

Niederwerder et al [11] used splenic homogenate from an ASFV infected pig in RPMI media to mix with feed, and in the current studies, we used serum from an ASFV infected pig mixed into commercially collected liquid porcine plasma. The different virus source may have impacted the different results between studies. For example, differences in stability of Classical swine fever virus from different tissues in pigs have been reported [17] and the stability differences were attributed to differences in enzymes, lipids and other components between different tissues. In the current studies the use of LPP as a media for the stock virus could have potentially impacted the capacity of ASFV to cause infection. Past studies have demonstrated that experimentally infected pigs provided spray-dried plasma (SDP) in feed had a more rapid clearance rate of the tested viruses [18, 19]. Furthermore, the enhanced virus clearance for pigs supplemented with SDP may have been expediated by a beneficial modulation of cytokines in the lung fluid [18,19]. However, it is unknown if SDP provided in feed could reduce incidence of ASFV transmission or expedite ASFV clearance in surviving infected pigs.

Little is known about ASFV transmission, albeit a few studies confirm that the MID is higher for the oral route compared to the intranasal one [20, 21]. Similarly, the MID is lower if the ASFV is delivered in drinking water compared to being mixed into the feed [11, 20, 22]or in liquid milk [23]. The results of our studies are consistent with epidemiological data that did not report a single case of ASFV caused by commercial feed as a source of infection, suggesting the MID for commercial feed is high [24].

In these current studies, since feeding ASFV contaminated LPP on feed did not cause infection, it was important to demonstrate if the ASFV contaminated LPP was infective. Therefore, following the feeding and observation periods of the current studies the pigs were then dosed with higher levels of the LPP and by alternative routes of exposure including IM injection and IG gavage. LPP containing serum from ASFV infected pigs could cause infection in both Studies. The pigs in Study I that were injected IM with 10 mL of ASFV inoculated LPP died 3 days after inoculation. The rapid development of high fever and mortality for the IM pigs was likely due to hyper-acute infection because of the high viral dose injected into the pig. Intramuscular injection of live virus is a more efficient exposure [25,26]. Similarly, in both studies I and II, pigs exposed to IG gavage of 50 mL of LPP containing serum from ASFV infected pigs became infected. Niederwerder et al. [11] reported that the MID of ASFV was much lower when provided in liquid RPMI as drinking water (10^0^ TCID_50_) than when provided on feed (10^4^ TCID_50_). The IG method used in our study would more likely mimic drinking since it delivers a more rapid dose of virus directly to the stomach compared to allowing the LPP to absorb into the feed and allowing the pigs to consume the feed naturally. However, the IG method may bypass potential sites of infection in the oro-pharynx, fact that does not occur when drinking liquids naturally. It is not possible to rule out that IM pigs in Study I could have transmitted ASFV by contact with the IG pigs because they were maintained in the same pen. For example, in Study II following the experimental feeding and observation period, only half of the pigs were IG gavaged while the remaining pigs served as sentinel pigs. One of the sentinel pigs became infected confirming the potential for pig to pig transmission. These results demonstrate that the LPP contaminated with serum from ASFV infected pigs contained infective ASFV capable of causing infection. The fact that LPP administered IG was infective but not when blended with commercial feed may suggest a longer time for the feed under the acid pH at the stomach may play a role in reducing the infectivity of contaminated commercial feed compared with liquid administration and also confirms the susceptibility of pigs to intragastric infection.

## Summary and conclusions

In these studies, commercially collected LPP was contaminated with serum from ASFV infected pigs representing 0.4% or 2.0% of the pigs being slaughtered would be infected, asymptomatic and passed antemortem inspection. Feed was mixed with the contaminated LPP and fed to pigs for 14 consecutive days providing a daily dose of ASFV 10^4.3^ or 10^5.0^ log TCID_50_/pig followed by a 5 or 9 days observation period (Studies I and II, respectively). In both experiments, pigs did not become infected demonstrating that the MID in feed from LPP containing serum from ASFV infected pigs was higher than 10^5.0^ log TCID_50_/pig. The recoverable ASFV titer in feed implied that there were no components in the commercial feed that significantly reduced the potential infectious capacity of the ASFV inoculum. These results suggest that unprocessed LPP may contain inherent properties that could diminish the infectious capacity of ASFV. It was concluded that under the experimental conditions used, the minimum infectious dose of ASFV in feed is higher than previously reported when pigs are repeatedly exposed to unprocessed ASFV inoculated liquid plasma mixed on feed.

## Supporting information

S1 Table Commercial feed composition used in the study. (DOCX)
