# Supplementary material for: Commercial feed containing porcine plasma spiked with African swine fever virus is not infective in pigs when administered for 14 consecutive days
Source: PLoS One. 2020 Jul 22;15(7):e0235895. doi: 10.1371/journal.pone.0235895 (PMC7375527; doi:10.1371/journal.pone.0235895)
Supplement: S1 Table — (DOCX) [file pone.0235895.s001.docx]

**S1_Table. Commercial feed composition used in the study.**

| Composition | |
| --- | --- |
| Barley, Corn, Wheat, Roasted soya extracted flour, Oats, Toasted soybeans, Sugar beet pulp, Animal fat, Dicalcium phosphate, Soy lecithin, Calcium carbonate, Sodium chloride, Butyric acid, L-Lysine, Methionine, L-Threonine, L-Tryptophan, L-Valine | |
| Vitamins and microelements: Vit. A (13.01 UI/kg), Vit. D3 (1.001 UI/kg), Vit. E (157.63 ppm), Choline chloride (138 ppm), 25-hydroxicolecalciferol (25.0 µg/kg), Iron sulphate (111 ppm), Iodine (1.0 ppm), Copper sulphate (2.5 ppm), Copper trihydroxychloride (100 ppm), Manganese oxide (31 ppm), Zinc oxide (61 ppm), Zinc hydrochloride (60 ppm), Selenium (0.15 ppm) | |
| Enzymes: Beta-glucanase, Alpha-amylase, Beta-xylanase, Phytase | |
| Flavoring: Sodium saccharine (164 ppm), Neohesperidine dihydrochalcone (0.8 ppm) | |
| Preservatives: Formic acid, sodium format, propionic acid | |
| Antioxidants: BHT (98.1 ppm), propyl gallate (7.6 ppm), citric acid | |
| Anti-binder agents: Natural mixture of steatites and chlorites, Kieselgur, Betnonite, sepiollite | |
| Stabilizer: Hydrolyzed lecithin | |
| Declared Analytical Components | |
| Crude Protein, % | 17.50 |
| Crude Fat, % | 4.50 |
| Crude Fiber, % | 4.17 |
| Ash, % | 4.58 |
| Lysine, % | 1.26 |
| Methionine, % | 0.48 |
| Calcium, % | 0.69 |
| Phosphorous, % | 0.51 |
| Sodium, % | 0.30 |
